# Increased dosage of the imprinted *Ascl2* gene restrains two key endocrine lineages of the mouse Placenta

**DOI:** 10.1016/j.ydbio.2016.08.014

**Published:** 2016-10-01

**Authors:** S.J. Tunster, G.I. McNamara, H.D.J. Creeth, R.M. John

**Affiliations:** Cardiff School of Biosciences, Cardiff University, Cardiff, Wales CF103AX, UK

**Keywords:** *Ascl2*, Placenta, *Phlda2*, Epigenetics, Imprinting

## Abstract

Imprinted genes are expressed primarily from one parental allele by virtue of a germ line epigenetic process. *Achaete-scute complex homolog 2* (*Ascl2* aka *Mash2*) is a maternally expressed imprinted gene that plays a key role in placental and intestinal development. Loss-of-function of *Ascl2* results in an expansion of the parietal trophoblast giant cell (P-TGC) lineage, an almost complete loss of *Trophoblast specific protein alpha* (*Tpbpa*) positive cells in the ectoplacental cone and embryonic failure by E10.5. *Tpbpa* expression marks the progenitors of some P-TGCs, two additional trophoblast giant cell lineages (spiral artery and canal), the spongiotrophoblast and the glycogen cell lineage. Using a transgenic model, here we show that elevated expression of *Ascl2* reduced the number of P-TGC cells by 40%. Elevated *Ascl2* also resulted in a marked loss of the spongiotrophoblast and a substantial mislocalisation of glycogen cells into the labyrinth. In addition, Ascl2-Tg placenta contained considerably more placental glycogen than wild type. Glycogen cells are normally located within the junctional zone in close contact with spongiotrophoblast cells, before migrating through the P-TGC layer into the maternal decidua late in gestation where their stores of glycogen are released. The failure of glycogen cells to release their stores of glycogen may explain both the inappropriate accumulation of glycogen and fetal growth restriction observed late in gestation in this model. In addition, using in a genetic cross we provide evidence that *Ascl2* requires the activity of a second maternally expressed imprinted gene, *Pleckstrin homology-like domain, family a, member 2* (*Phlda2*) to limit the expansion of the spongiotrophoblast. This “belts and braces” approach demonstrates the importance of genomic imprinting in regulating the size of the placental endocrine compartment for optimal placental development and fetal growth.

## Highlights

• Increased Ascl2 dosage results in a gross mislocalisation of placental glycogen cells and fetal growth restriction.
• *Ascl2* restrains expansion of the parietal trophoblast giant cell lineage
• *Ascl2* acts upstream of *Phlda2* to restrain expansion of the spongiotrophoblast.
• These data highlight a “belts and braces” approach to regulating placental lineage development.

## 1 Introduction

Genomic imprinting is a remarkable process whereby certain genes are preferentially silenced on one parental allele as a consequence of epigenetic events initiated in the germ line (Surani, 1998). Imprinted genes have been shown to functionally converge on biological processes that, while not exclusive to mammals, were likely important for the relative dominance of mammals on Earth today, including placentation, advanced maturity at birth and high maternal care (Kaneko-Ishino and Ishino, 2010, Keverne, 2013, Renfree et al., 2013, Moore, 2011, Cleaton et al., 2014, Peters, 2014). The majority of studies on imprinted genes rely on loss-of-expression models. However, imprinting is a mechanism that exclusively modulates gene dosage. Examining models in which gene dosage is increased may further contribute to our understanding of genomic imprinting.

*Achaete-scute complex homolog 2* (*Ascl2* aka *Mash2*) was one of the first imprinted genes to be knocked out in mice (Guillemot et al., 1995). These studies demonstrated that fetal survival beyond E10.5 requires the maternal *Ascl2* allele (Guillemot et al., 1995, Guillemot et al., 1994). Loss of function restricted to the embryo had no overt consequence during gestation highlighting a requirement for placental *Ascl2* in the transition to the mature chorioallantoic placenta (Tanaka et al., 1997). The mature mouse placenta is organised into the histologically distinct labyrinth zone, junctional zone and maternal decidua, all of which are interspersed with trophoblast giant cells (TGCs) (John and Hemberger, 2012, Rai and Cross, 2014). Loss-of-function of *Ascl2* resulted in an expansion of one TGC type, the parietal (P-) TGCs. These normally form a single layer of cells located beneath the maternal decidua surrounding large lakes of maternal blood leading into the uterine veins, that becomes somewhat discontinuous as development proceeds (Rai and Cross, 2014). In *Ascl2*^−/+^ placenta there were multiple layers of P-TGCs (Guillemot et al., 1994, Tanaka et al., 1997). Four other distinct TGC types exist which are defined by their characteristic gene expression profiles and their position with respect to maternal circulation (Rai and Cross, 2014, Simmons et al., 2007, Simmons et al., 2008, Gasperowicz et al., 2013). The spiral artery (SpA-) TGCs are located within the maternal decidua lining the maternal blood system on entry to the placenta; the canal (C-) TGCs line the maternal blood canals as they pass through the junctional zone; the sinusoidal TGCs (S-TGCs) are in direct contact with the maternal blood spaces within the labyrinth and the recently discovered channel (Ch-) TGCs line the maternal blood spaces located just beneath the decidua where maternal blood drains into the venous sinuses to be returned to the maternal circulation (Rai and Cross, 2014, Simmons et al., 2007, Simmons et al., 2008, Gasperowicz et al., 2013). These additional lineages were not examined in *Ascl2*^−/+^ placenta as these fail too early in development, but the original report (Guillemot et al., 1994) noted an almost complete lack of expression of *Trophoblast specific protein alpha (Tpbpa)*. *Tpbpa* is expressed in the progenitor cells of 50% of the P-TGCs, all the SpA-TGCs and C-TGCs, as well as the glycogen cell and spongiotrophoblast lineages that form the bulk of the junctional zone (Hu and Cross, 2011). Another mouse model (*Del*^*7A1/+*^) in which *Ascl2* levels were reduced by 50%, allowed survival to term and the assessment of a later placental phenotype (Oh-McGinnis et al., 2011). *Del*^*7A1/+*^ placenta possessed a similarly expanded P-TGC layer alongside a reduced spongiotrophoblast and a complete loss of the glycogen cell lineage (Oh-McGinnis et al., 2011, Lefebvre et al., 2009). *Del*^*7A1/+*^ involves maternal inheritance of a 280 kb deletion physically adjacent to *Ascl2* and spanning the maternally expressed *Tyrosine hydroxylase* (*Th*) gene. Consequently, these placenta lack *Th* expression after maternal transmission of this deletion. *Del*^*7A1/+*^ placenta also express *Pleckstrin Homology-Like Domain, Family A, Member 2* (*Phlda2*) at two-fold the normal level (Data summarised in Supplemental Table 1). *Phlda2* maps to the same imprinted domain at *Ascl2* and *Th* (Fitzpatrick et al., 2002). We have previously shown that two-fold expression of *Phlda2* results in a 50% loss of the spongiotrophoblast lineage (Tunster et al., 2010, Tunster et al., 2014, Tunster et al., 2015). Consequently the loss of spongiotrophoblast in the *Del*^*7A1/+*^ placenta may be a direct consequence of reduced *Ascl2* expression or increased *Phlda2* expression.

These *in vivo* studies highlighted a potential role for *Ascl2* as a dosage-sensitive cellular switch between the spongiotrophoblast/glycogen cell lineages and the P-TGC lineage. Consistent with a role in lineage choice, *Ascl2* is expressed in the ectoplacental cone and extra embryonic ectoderm at E7.5 where the placental progenitors reside with expression restricted to the diploid trophoblast cells, and later diploid trophoblast cells located in the labyrinthine and spongiotrophoblast layers (Guillemot et al., 1994, Guillemot et al., 1993). This model would be consistent with the finding that ectopic expression of *Ascl2* can inhibit the normal differentiation of Rcho-1 cells, a rat choriocarcinoma cell line, towards a TGC-like fate (Kraut et al., 1998, Cross et al., 1995, Hughes et al., 2004). However, ectopic expression of *Ascl2* in trophoblast stem (TS) cells, which can differentiate into a number of trophoblast lineages, resulted in decreased expression of both *Prl3d*, a marker of TGCs, and *Tpbpa*, a gene expressed in the progenitors of a number of lineages including the spongiotrophoblast, suggesting that *Ascl2* could function to repress more than one placental lineage (Hughes et al., 2004, Takao et al., 2012). The consequences of increased expression of *Ascl2 in vivo* on placental development have not been fully explored. A P1 transgene spanning the genomic locus expressing *Ascl2* at 4–7 fold the endogenous level was shown to rescue embryonic lethality associated with maternal inheritance of the *Ascl2* targeted allele, but no overt consequence for placental or fetal weight were reported (Rentsendorj et al., 2010). To investigate the effect of *Ascl2* overexpression on placental development and fetal growth, we made use of an existing line of mice carrying a bacterial artificial chromosome (BAC) spanning the *Ascl2* locus previously used to explore the relevance of elevated *Ascl2* in intestinal tumorigenesis (Reed et al., 2012). We observed a reciprocal relationship between *Ascl2* expression and P-TGC number as anticipated, but a marked loss of spongiotrophoblast arguing against a role for *Ascl2* as a simple cell fate switch.

## 2 Materials and methods

### 2.1 Mouse strains and genotyping

All animal studies and breeding was approved by the Universities of Cardiff ethical committee and performed under a UK Home Office project license (RMJ). Mice were housed in a conventional unit on a 12-h light–dark cycle with lights coming on at 06.00 h with a temperature range of 21 °C ±2 with free access to tap water and standard chow unless otherwise stated. The *Ascl2* BAC transgenic line (Ascl2-Tg; Genome Systems BAC225J16) described previously (Reed et al., 2012) was primarily studied on the C57BL/6 (BL6) strain background. Some experiments were performed when the transgene was bred into 129S2/SvHsd (G6; 129). The *Phlda2* targeted allele (Frank et al., 2002) was maintained by paternal transmission on the BL6 genetic background.

### 2.2 Weighing studies

Embryonic and placental wet weights were taken following careful dissection to remove the yolk sac, umbilicus and excess decidua prior to RNA extraction at the stated time points after a discernable plug. Genotyping data was obtained from yolk sac DNA using primers CACATACGTTCCGCCATTCC and CCACTTCAACGTAACACCGC to amplify from the BAC.

### 2.3 Quantitative RNA analysis

Quantitative PCR of reverse transcribed RNA (QRT-PCR) was performed as described (Tunster et al., 2010) with additional primers (Supplemental Table 2).

### 2.4 *In situ* hybridisation and histological analyses

Placentas were fixed overnight in phosphate-buffered 4% paraformaldehyde, paraffin-embedded and 10 µm sections taken through the midline. Haematoxylin and eosin (H&E) staining, riboprobe preparation, *in situ* hybridizations and PAS staining were performed as previously described (Tunster et al., 2010, John et al., 2001). Biochemical determination of glycogen was performed on fresh snap-frozen placenta as previously described (Tunster et al., 2010, John et al., 2001). Measurements were taken from midline sections obtained by cutting a number of serial sections and judging overall thickness as previously described (Tunster et al., 2010). For counting P-TGC, sections were analysed without knowing the genotype, with independent repeat blind counts.

### 2.5 Statistical analyses

Statistical significance (Probability values) was determined using the Student's *t*-Test (two tailed distribution and two sample unequal variance). The significance in the difference in observed over the expected appearance of a particular genotype was determined using the *Chi*-squared Test.

## 3 Results

**Fig. 1:**
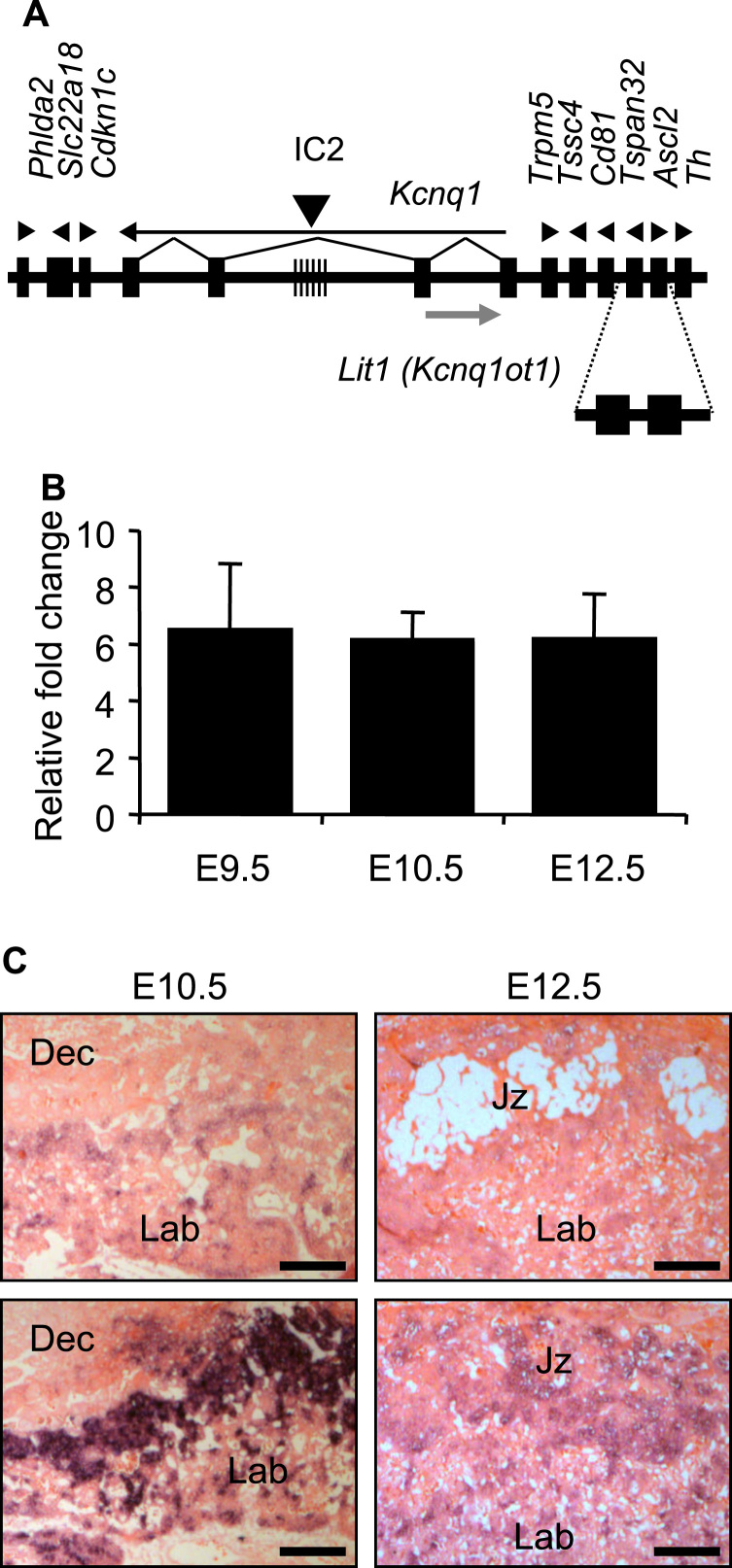
Spatially and temporally accurate (entopic) overexpression of *Ascl2* in the mouse placenta. (A) Schematic of the BAC transgene. Top line shows a genomic map of the IC2 region on distal mouse chromosome 7. Hatched region marks the *KvDMR1* that is methylated only on the maternal allele. Arrows indicate direction of transcription. Below is a map of the BAC transgene containing the *Ascl2* gene. Filled boxes represent imprinted genes (not to scale). (B) Placental RT-QPCR results for *Ascl2* in transgenic placenta at E9.5, E10.5 and E12.5. N=4 placenta per genotype (2 *versus* 2 from 2 independent litters); error bars represent SEM. Statistical significance calculated using *t*-test. **P<0.01, ***P<0.005. (C) *In situ* hybridisation of midline placental sections with *Ascl2* riboprobe reveals similar spatial localisation of mRNA in control and Ascl2-Tg placentas at E10.5 and E12.5. Scale bars=200 µm. Data for Fig. 2 in Supplemental Table 2.

The previously described transgenic line (Reed et al., 2012) carries a bacterial artificial chromosome physically encompassing the *Ascl2* locus (Fig. 1A). E9.5, E10.5 and E12.5 Ascl2-Tg transgenic placenta were found to express *Ascl2* at ~6-fold the wild type level (Fig. 1B, Supplemental Table 3). The transgene spanned a second gene, *Tspan32*, not expressed in the placenta (Gartlan et al., 2010, Nicholson et al., 2000) and not ectopically expressed from the transgene (Supplemental Fig. 1). *In situ* hybridisation with an *Ascl2* riboprobe demonstrated the correct localisation of the *Ascl2* signal within Ascl2-Tg placenta to a subset of diploid cells in the ectoplacental cone and chorion at E10.5 and restricted expression within a subset of cells within the junctional zone at E12.5 (Fig. 1C). Previously, adenovirus driven overexpression of *Ascl2* at 10-fold the normal level was found to down-regulate expression of both *Tpbpa* and *Prl3d1* in trophoblast stem cells cultured under stem cell conditions, alongside reduced expression of *Phlda2* (Takao et al., 2012) (Supplemental Table 1). The spatio-temporally accurate elevated expression of *Ascl2* in this animal model provided a tool to explore the consequences of similarly increased *Ascl2* dosage on placental development *in vivo*.

**Fig. 2:**
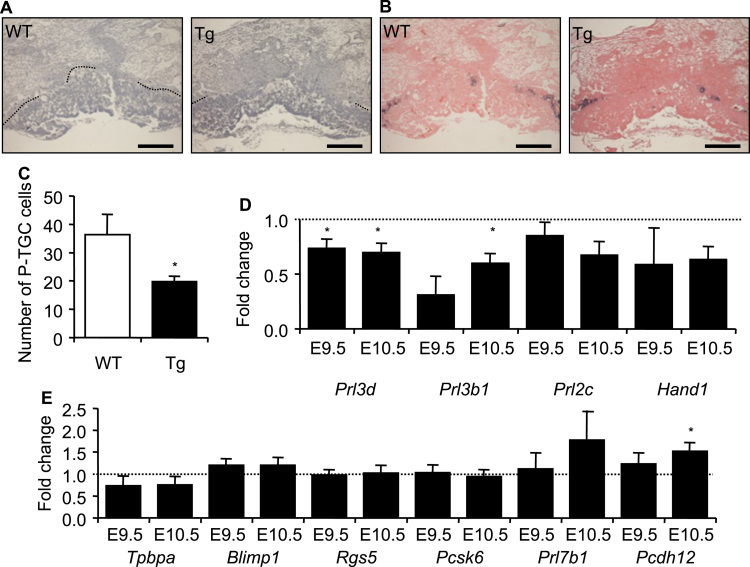
A reduction in the parietal trophoblast giant cell lineage. (A) Haematoxylin staining of midline placental sections at E10.5. Parietal trophoblast giant cells (P-TGC) are indicated by dashed lines. Scale bars=500 µm. (B) *In situ* hybridisation with a *Prl3d* (*Pl1*) riboprobe, which specifically marks P-TGCs at E10.5. Scale bars=500 µm. (C) P-TGC number per section at E10.5. N=26. (D) Relative expression levels of key markers of P-TGC at E9.5 and E10.5. *Prl3d1, Prl3b1* (aka *Pl2*), *Prl2c* (aka *Plf*) and *Hand1* are all expressed the P-TGC lineage at this time point. (E) Relative expression levels of markers of the emerging spongiotrophoblast (*Tpbpa*), glycogen cell (*Pcdh12, Prl7b1*), S-TGC, SpA-TGC and C-TGC (*Blimp1*, *Rgs5*, *Pcsk6*, *Prl7b1*) lineages at E9.5 and E10.5. For the RT-qPCR analysis, N=4 placenta per genotype (2 *versus* 2 from 2 independent litters); error bars represent SEM. Statistical significance calculated using *t*-test. ^NS^*P*>0.05, *P<0.05. Data for Fig. 2 in Supplemental Table 3.

At E10.5, P-TGC form a distinct and histologically recognisable single cell layer between the maternal decidua and the developing chorio-allantoic placenta. Haematoxylin staining of E10.5 Ascl2-Tg midline placental sections revealed a marked loss of the giant, polyploid cells lining the maternal decidua (Fig. 2A). *In situ* hybridisation with *Prl3d* (*Pl1*), exclusively expressed in the P-TGC lineage at this time point (Jackson et al., 1986) identified these polyploid cells as P-TGCs (Fig. 2B). Counting of these cells in midline sections revealed a significant ~40% loss of P-TGCs at E10.5 (36.3±7.1 *versus* 19.8±1.9; 10 WT v 16 Ascl2-Tg placental sections from 5 litters; *p*=0.0120; Fig. 2C, Supplemental Table 4). Consistent with the loss of P-TGCs, RT-QPCR revealed a significant 30% reduction in expression of *Prl3d* at E9.5 and E10.5 (Fig. 2D). Expression of *Prl3b1* (aka *Pl2*), a second gene expressed in P-TGC at E9.5 and E10.5 (Simmons et al., 2008), was also markedly reduced, by 70% and 40% respectively (Fig. 2D, Supplemental Table 4). *Hand1* and *Prl2c,* genes expressed in all or most of the TGC lineages (Simmons et al., 2008, Scott et al., 2000), were not significantly altered suggesting the defect was not attributable to the loss of other emergent TGC lineages. The loss of P-TGC was reciprocal to the phenotype reported in response to reduced *Ascl2* expression (Guillemot et al., 1994, Tanaka et al., 1997). Markers associated with the emerging spongiotrophoblast and glycogen cell lineages were also measured (Fig. 2E). *Tpbpa,* expressed in the precursors of the spongiotrophoblast, the glycogen cells, S-TGC, SpA-TGC and 50% of the P-TGC (Hu and Cross, 2011), was expressed at near normal levels (Fig. 2E). *Blimp1, Rgs5, Pcsk6* and *Prl7b1*, expressed in the precursors of the glycogen cells, the SpA-TGC and the C-TGC (Mould et al., 2012) or exclusively in the SpA TGC (Mould et al., 2012) were expressed at near normal levels. *Protocadherin 12* (*Pcdh12*), one of the earliest markers of the glycogen cell lineage (Bouillot et al., 2006), was expressed at wild type levels at E9.5 but elevated at E10.5, by 1.5-fold (Fig. 2E, Supplemental Table 4).

**Fig. 3:**
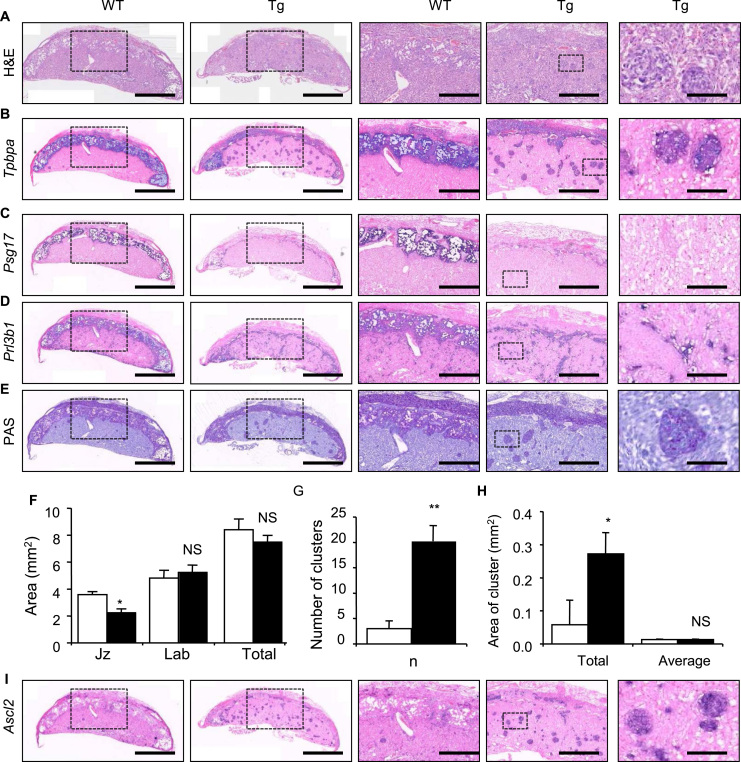
A substantial loss of the spongiotrophoblast and mislocalisation of glycogen cells at E14.5. (A) H+E staining at E14.5 illustrating loss of the junctional zone. (B) *In situ* hybridisation with a *Tpbpa* riboprobe, which specifically marks the spongiotrophoblast and glycogen cell lineages in the junctional zone, at E14.5. (C) *In situ* hybridisation with a *Psg17* riboprobe, which is expressed in just the spongiotrophoblast at E14.5. (D) *In situ* hybridisation with a *Prl3b1* riboprobe, which is expressed in the P-TGC, C-TGC, S-TGC and the spongiotrophoblast at E14.5, showing a degraded interface between the junctional zone and maternal decidua. (E) PAS staining of glycogen cells in the junctional zone at E14.5. Three right hand images highlighting mislocalisation of glycogen cells in the labyrinth. (F) Midline section areas (mm^2^) occupied by the junctional zone and labyrinth, and total area of both at E14.5. (G) Number of *Tpbpa*+ve/*Psg17*−ve/*Prl3b1*−ve/PAS+ve/*Ascl2*+ve clusters present in the labyrinth. (H) Total and average area (mm^2^) occupied by *Tpbpa*+ve/*Psg17*−ve/*Prl3b1*−ve/PAS+ve/*Ascl2*+ve clusters in the labyrinth. (I) *In situ* hybridisation with an *Ascl2* riboprobe, demonstrating mislocalised cells express *Ascl2*. Scale bars=1000 µm (two images on left); 350 µm (middle and adjacent right image) and 150 µm (far right image).

To further investigate the effects of the *Ascl2* transgene on the placental lineages, Ascl2-Tg placenta were histologically examined at E14.5. H&E staining revealed an unexpected, distinct and dramatic loss of the junctional zone, where the glycogen and spongiotrophoblast cells normally reside (Fig. 3A). *In situ* hybridisation with *Tpbpa,* exclusively expressed in both the spongiotrophoblast and the glycogen cells (Lescisin et al., 1988), further highlighted the dramatic loss of cells from the junctional zone (Fig. 3B). *In situ* hybridisation with *Psg17*, an exclusive marker of the spongiotrophoblast (Kromer et al., 1996), suggested a substantial loss of this lineage from the junctional zone (Fig. 3C). *In situ hybridisation* with *Prl3b1*, expressed in the spongiotrophoblast, C-TGC, P-TGC and S-TGCs at E14.5 (Simmons et al., 2008), was also consistent with a significant loss of the spongiotrophoblast lineage from the junctional zone, and further identified a degradation of the interface between the junctional zone and maternal decidua consistent with the loss of P-TGC from this region (Fig. 3D). What little remained of the junctional zone stained for Periodic acid Schiff (PAS), which distinguishes glycogen cells (Adamson et al., 2002), suggesting that the junctional zone was primarily composed of this cell type (Fig. 3E). There was an overall 40% decrease in junctional zone area (Fig. 3F; Supplemental Table 5) and an increased number of junctional zone-like clusters of cells present in the labyrinth. While clusters were apparent in both WT and Ascl2-Tg placenta, the number present in the labyrinth of Ascl2-Tg placenta was 7-fold greater than WT (Fig. 3G; Supplemental Table 5). The size of the clusters was similar between the two genotypes (Fig. 3H; Supplemental Table 5) and they were *Tpbpa*+ve/*Psg17*−ve/*Prl3b1*−ve/*Ascl2*+ve/PAS+ve (Fig. 3B–E, I; right panels). This analysis identified them as mislocalised glycogen cells.

**Fig. 4:**
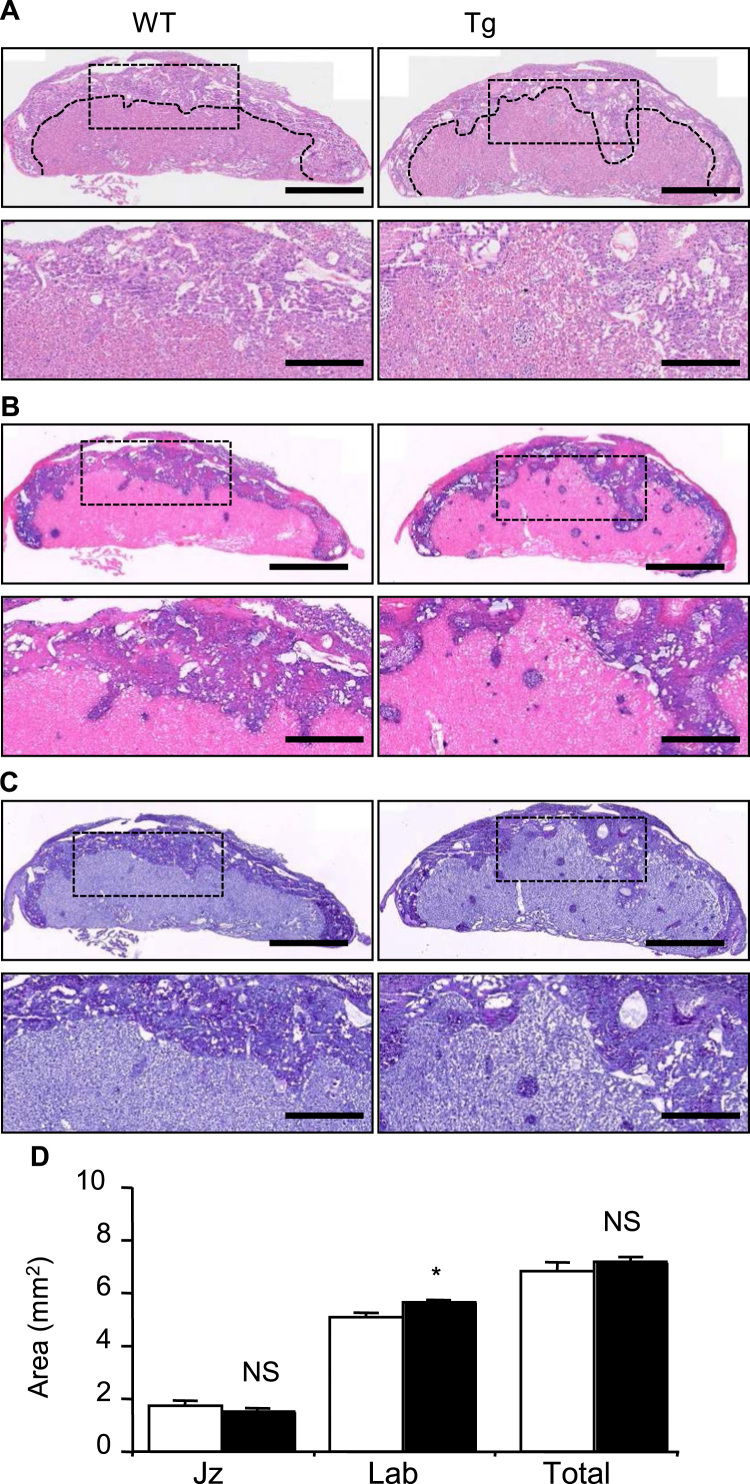
Persistence of junctional zone phenotype at E18.5. (A) H+E staining at E18.5 illustrating loss of the junctional zone. Lower panels are higher magnification of the regions indicated. (B) *In situ* hybridisation with a *Tpbpa* riboprobe, which specifically marks the spongiotrophoblast and glycogen cell lineages, at E18.5. Lower panels are higher magnification of the regions indicated. (C) PAS staining for glycogen cells at E18.5. Lower panels are higher magnification of the regions indicated. (D) Midline section areas (mm^2^) occupied by the junctional zone and labyrinth, and total area of both at E18.5. Scale bars A–C=1000 µm (upper panels); 400 µm (lower panels).

At E18.5, just prior to term, the junctional zone was similarly disorganised with clusters of mislocalised glycogen cells observable in the labyrinth of Ascl2-Tg placentae (Fig. 4A–C). As quantified by area measurements of midline sections, the 12% decrease in junctional zone at E18.5 was not significantly different to WT and there was small (11%) but significant increase in labyrinth (Fig. 4D, Supplemental Table 6).

**Fig. 5:**
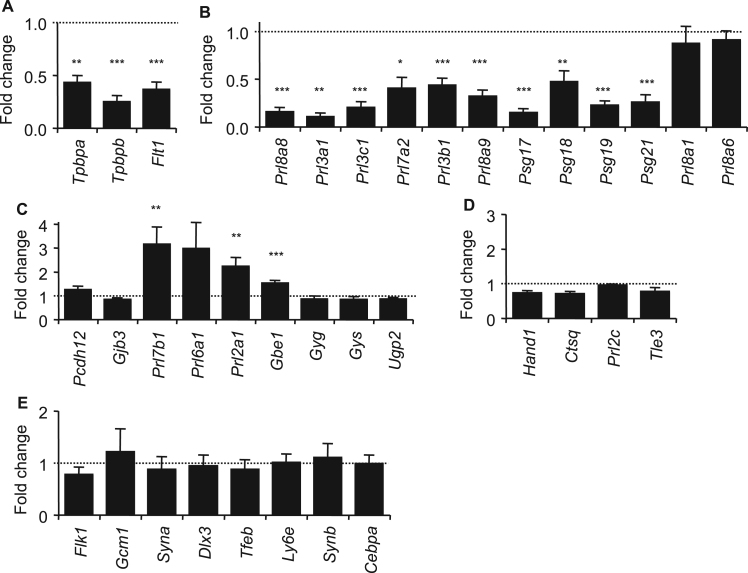
Lineage analysis reveals a marked loss of the spongiotrophoblast and an increased representation of the glycogen cell lineage. (A) Quantitation of junctional zone markers *Tpbpa, Tpbpb* and *Flt1* at E14.5. (B) Quantitation of genes exclusively or predominantly expressed in the spongiotrophoblast at E14.5. (C) Quantitation of genes exclusively or predominantly expressed in the glycogen cell lineage at E14.5. (D) Quantitation of genes exclusively or predominantly expressed in the TGC lineages at E14.5. (E) Quantitation of genes exclusively or predominantly expressed in the labyrinth at E14.5. For the RT-qPCR analysis, N=4 placenta per genotype (2 vs. 2 from 2 independent litters); error bars represent SEM. Statistical significance calculated using *t*-test. ^NS^*P*>0.05, *P<0.05, **P<0.01, ***P<0.005. Data for Fig. 5 in Supplemental Table 4.

As a proxy for the representation of the different lineages, a RT-QPCR analysis was performed (Fig. 5, Supplemental Table 7). *Tpbpa, Tpbpb* and *FMS-like tyrosine kinase 1* (*Flt1*), markers of the junctional zone lineages at E14.5 (Henke et al., 2013, Hirashima et al., 2003), were expressed at considerably lower levels in Ascl2-Tg placenta compared to controls (Fig. 5A). *Prl8a8*, *Prl3a1*, *Prl3c1*, *Prl3a1, Prl7a2*, *Prl8a9*, *Psg17*, *Psg18*, *Psg19* and *Psg21*, all markers exclusively or predominantly expressed in the spongiotrophoblast (Simmons et al., 2008, McLellan et al., 2005), were expressed at markedly lower levels in Ascl2-Tg placenta (Fig. 5B). *Prl3b1,* which at E14.5 is expressed in P-TGC, C-TGC, S-TGC and spongiotrophoblast (Simmons et al., 2008), was reduced (Fig. 5B). However two other markers associated predominantly with the spongiotrophoblast, *Prl8a1* (SpT and P-TGCs) and *Prl8a6* (SpT and C-TGCs) (Simmons et al., 2008), were expressed at wild type levels. These data essentially supported a loss of the spongiotrophoblast lineage.

Markers of the glycogen cell lineage were also assessed (Fig. 5C). *Pcdh12* and G*ap junction protein, beta 3* (*Gjb3*/*Cx31*), a marker of glycogen cell maturation (Zheng-Fischhofer et al., 2007), were expressed at near normal levels, however *Prl7b1* and *Prl2a1*, expressed predominantly but not exclusively in glycogen cells (Simmons et al., 2008), were both significantly elevated (Fig. 5C). *Prl7b1* is a marker of migrating glycogen cells and is also expressed in SpA-TGC and C-TGC at E14.5, while *Prl2a1* is expressed in P-TGC, SpA-TGC and C-TGC, in addition to the glycogen cell lineage (Simmons et al., 2008). *Glucan (1,4-alpha-), branching enzyme 1* (*Gbe1*), involved in glycogen branching, was elevated. However, *Prl6a1*, expressed in glycogen cells and SpA-TGCs, and other glycogen metabolism enzymes, *glycogenin* (*Gyg*), *glycogen synthase 1* (*gys*) and *UDP-glucose pyrophosphorylase 2* (*Ugp2*), were not significantly elevated. These data supported a change in the nature of the glycogen cell lineage rather than a significant alteration in the cellular contribution of this lineage to the placenta.

An analysis of the markers *Hand1, Tle3* and *Prl2c,* genes expressed in all or most of the TGC lineages (Simmons et al., 2008, Gasperowicz et al., 2013, Scott et al., 2000), and *Ctsq,* expressed in just the S-TGC and Ch-TGC lineages (Rai and Cross, 2014), suggested a near normal representation of these lineages (Fig. 5D). *Flk1*, *Dlx3*, *Tfeb*, *Syna*, *Synb*, *Gcm1* and *Cebpa*, all genes primarily or exclusively expressed in the labyrinth at E14.5 (Simmons et al., 2008, Hirashima et al., 2003, Steingrimsson et al., 1998, Berghorn et al., 2005, Anson-Cartwright et al., 2000) were also expressed at levels similar to controls (Fig. 5E). Together, these data were consistent with a substantial loss of the spongiotrophoblast and P-TGCs with limited consequence for the other placental lineages.

**Fig. 6:**
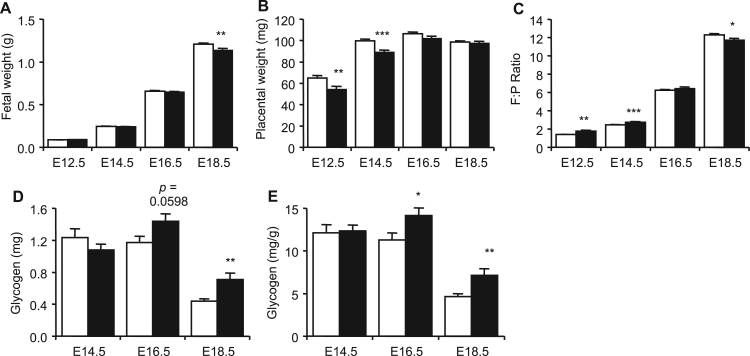
Placental and fetal growth restriction but increased placental glycogen. (A) Transgenic *versus* non-transgenic fetal wet weights at E12.5 (WT N=29; Ascl2-Tg N=19), E14.5 (WT N=42; Ascl2-Tg N=40), E16.5 (WT N =25; Ascl2-Tg N=28) and E18.5 (WT N=41; Ascl2-Tg N =33). (B) Transgenic *versus* wild type placental wet weights at the same time points. (C) Fetal:Placental (F:P) ratios at the same time points. (D) Biochemical determination of placental glycogen at E14.5, E16.5 and E18.5 expressed as the total amount of glycogen (mg) E. Biochemical determination of placental glycogen at E14.5, E16.5 and E18.5 expressed in relation to placental weight (mg/g). Data for Fig. 6 in Supplemental Table 5.

The loss of spongiotrophoblast was very marked in the Ascl2-Tg placenta. Previously we have shown a direct relationship between the spongiotrophoblast and the amount of stored placental glycogen late in gestation, both of which were associated with fetal growth restriction (Tunster et al., 2010, Tunster et al., 2014, Tunster et al., 2015, Salas et al., 2004). To assess the consequences of elevated *Ascl2*, fetuses and placenta were collected at E12.5, E14.5, E16.5 and E18.5. The observed ratios between Ascl2-Tg and non-transgenic fetuses were not significantly different to the expected ratios indicating no loss of viability (Critical value 3.841 with *p*=0.05 and DoF=1; E12.5: 2.083, E14.5: 0.049, E16.5: 0.170 and E18.5: 0.865). Ascl2-Tg fetuses were not significantly different in weight to controls at E12.5, E14.5, and E16.5 but at E18.5 there was a 6% (*p*=0.00521) reduction in wet weight (Fig. 6A, Supplemental Table 8). Ascl2-Tg placentae were significantly lighter than controls at E12.5 and E14.5, by 17% (*p*=0.00898) and 11% (*p*=9.29×10^−5^) respectively, but weights had recovered at E16.5 and E18.5 (Fig. 6B). As a consequence, the Fetal:Placental (F:P) ratios were higher at E12.5 and E14.5 and lower at E18.5 (Fig. 6C). A biochemical determination of glycogen was performed at E14.5, E16.5 and E18.5. At E18.5, there was a substantial (+61%; mg) increase in the total amount of glycogen present in Ascl2-Tg placenta (Fig. 6D). When expressed relative to placental weight (mg/g), glycogen was significantly increased at both E16.5 (+25%; *p*=0.0458) and E18.5 (+58%; *p*=0.00277).

Fetal growth restriction was very modest (6%) in comparison to the placental defect. Our analysis of a different mouse model (transgenic overexpression of *Phlda2*) with a similar substantial loss of spongiotrophoblast revealed a fetal growth restriction phenotype apparent on a 129 strain background and absent on a BL6 background (Tunster et al., 2010, Tunster et al., 2014, Tunster et al., 2015) potentially explained by the less permissive F:P ratio in 129 mice (Tunster et al., 2012). Ascl2-Tg was examined after >6 generations of backcrossing into 129. In initial studies, few plugged females were pregnant when checked at E14.5 (Supplemental Table 9). When the line was switched to a more nutrient-rich diet in a barrier unit, pregnancy success rates improved (Supplemental Table 9) but there was no difference in fetal weight between Ascl2-Tg fetuses and WT littermates at E18.5 under these more favourable conditions (Supplemental Table 10).

**Fig. 7:**
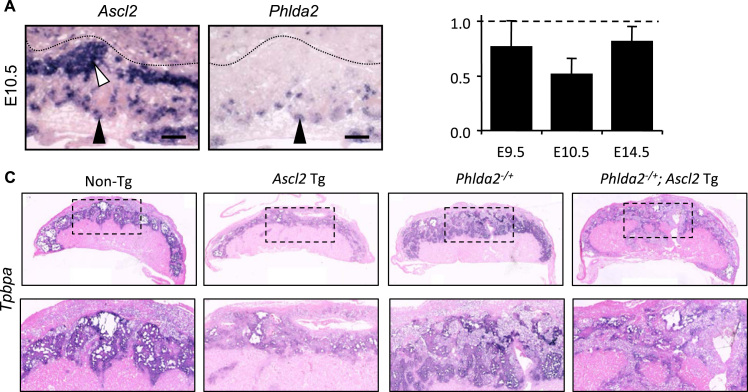
*Ascl2* requires *Phlda2* to repress the expansion of the spongiotrophoblast lineage. (A) *In situ* hybridisation of sequential midline sections of wild type placenta at E10.5 with *Ascl2* and *Phlda2* riboprobes. The two genes are co-expressed in subset of cells at the base of the developing junctional zone (filled arrowheads) but not those located near the P-TGC layer (white arrow). Scale bars 100 µm. (B) Quantitation of *Phlda2* mRNA at E9.5, E10.5 and E14.5. (C) *In situ* hybridisation of midline sections of non-transgenic, Ascl2-Tg, *Phlda2*^−/+^ and *Phlda2*^−^^/+^; Ascl2-Tg double transgenic placenta with the junctional zone marker *Tpbpa* at E14.5. D. *In situ* hybridisation of midline sections of non-transgenic, Ascl2-Tg, *Phlda2*^−/+^ and *Phlda2*^−/+^; Ascl2-Tg double transgenic placenta with an *Ascl2* riboprobe at E14.5. Scale bars in C and D =1000 µm (upper panels); 400 µm (lower panels).

**Fig. 8:**
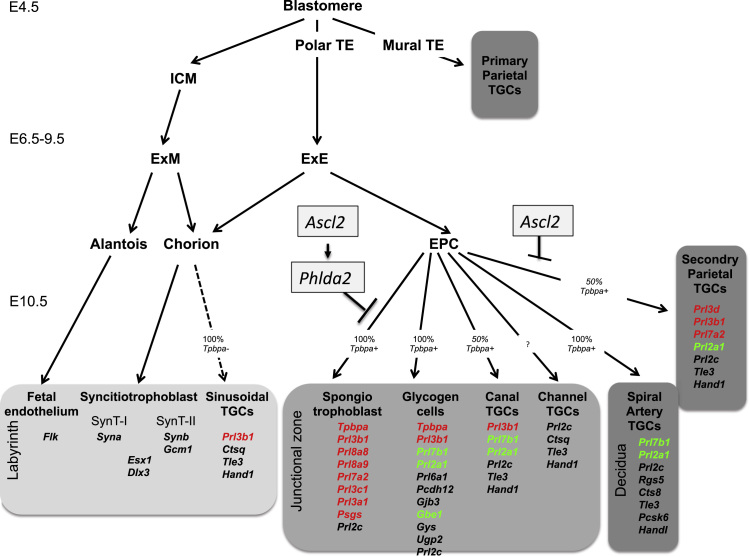
Summary of gene expression data in relation to placental lineages. Genes indicated in red are significantly down and genes indicated in green are significantly elevated in Ascl2-Tg placenta. *Ascl2* requires *Phlda2* to repress the expansion of the spongiotrophoblast lineage.

The dramatic reduction of the spongiotrophoblast lineage was consistent with data demonstrating that ectopic expression of *Ascl2* in TS cells represses the expression of *Tpbpa* (Takao et al., 2012) (Supplemental Table 1). However, a significant loss of the spongiotrophoblast lineage *in vivo* has been reported in association with a 50% reduction in the expression of *Ascl2* in *Del*^*7A1/+*^ placenta (Oh-McGinnis et al., 2011). In this complex model, *Phlda2* was expressed at two-fold higher than normal (Supplemental Table 1). We have previously shown that two-fold elevation in the expression of *Phlda2* results in a substantial (50%) reduction of the spongiotrophoblast (Tunster et al., 2010, Tunster et al., 2014, Tunster et al., 2015). Together these data suggest that *Ascl2* could regulate the spongiotrophoblast indirectly *via Phlda2. Phlda2* and *Ascl2* co-localise to a subset of cells with the developing chorio-allantoic placenta potentially marking progenitors of the spongiotrophoblast (Oh-McGinnis et al., 2011, Takao et al., 2012) (Fig. 7A). Ectopic expression of *Ascl2* in TS cells results in lower expression of *Phlda2* (Takao et al., 2012) (Supplemental Table 1). *In vivo*, a similar elevation in *Ascl2* did not result in significantly lower expression of *Phlda2* (Fig. 7B). To genetically test the relationship between *Ascl2* and *Phlda2*, double transgenic placenta carrying both the *Ascl2* transgene and a maternally inherited targeted *Phlda2* allele (*Phlda2*^*-/+*^*;*Ascl2-Tg) were generated. Both the *Phlda2* loss-of-function placenta and the double transgenic placenta possessed a markedly expanded junctional zone, as evidenced by *Tpbpa* staining of E14.5 placental sections (Fig. 7C). In the absence of *Phlda2*, *Ascl2* was detectable by *in situ* indicating that *Ascl2* expression was not dependent on *Phlda2* (Fig. 7D). While is possible that the dominance of the *Phlda2* phenotype is a consequence of earlier events, these data are consistent with *Ascl2* acting *via Phlda2* to suppress the expansion of the spongiotrophoblast lineage (Fig. 8).

## 4 Discussion

*Ascl2* is a gene expressed from the maternal allele in the placenta. Previous studies in mice examining the consequences of reduced expression of *Ascl2* have suggested a pivotal role for this gene in repressing the expansion of the parietal trophoblast giant cell lineage (Guillemot et al., 1995, Guillemot et al., 1994, Tanaka et al., 1997, Oh-McGinnis et al., 2011). Here, we have confirmed this directly in an *Ascl2* over expression model. We also highlight a novel role for the *Ascl2* in repressing the expansion of the spongiotrophoblast, a function that depends on expression of a second maternally expressed imprinted gene, *Phlda2*. We previously reported that two-fold expression of *Phlda2* resulted in a loss of the spongiotrophoblast associated with a significant reduction in placental glycogen, which led us to hypothesise a role for the spongiotrophoblast in driving the accumulation of these stores (Tunster et al., 2010). Here, we observed a similar loss of spongiotrophoblast but co-incident with increased stores of placental glycogen late in gestation. Moreover, glycogen cells were markedly mislocalised to the labyrinth. One interpretation of this data is that the mislocalisation of the glycogen cells, in response to the loss of both the P-TGC and the spongiotrophoblast, precludes the utilisation of these stores resulting in late fetal growth restriction.

We have now identified three maternally expressed genes located in a single, mechanistically distinct imprinted domain (Fitzpatrick et al., 2002) that all act on the spongiotrophoblast lineage of the mouse placenta. *Ascl2* and *Phlda2* repress the expansion of this lineage while *Cdkn1c* is required for this lineage to develop normally (Tunster et al., 2010, Tunster et al., 2014, Tunster et al., 2015, Salas et al., 2004, Tunster et al., 2011). Additionally, *Ascl2* and *Cdkn1c* are functionally important for the P-TGC and S-TGCs lineages, respectively. There is some evidence that the IC2 imprinted domain encompassing these genes became imprinted after marsupials diverged from Eutherian mammals (Suzuki et al., 2005, Suzuki et al., 2011). It may be significant that a key difference between marsupials and Eutherians is the extent to which extra embryonic tissues support growth *in utero* with the Eutherian newborn being substantially larger at term, relative to the size of the mother, and distinctly more mature than the marsupial newborn. There are a number of maternally expressed imprinted genes, including *Esx1*, *Cited1, Plac1* and *Nrk* (maternally expressed by virtue of their location on the paternally inactive X-chromosome), that repress the spongiotrophoblast lineage and at least two paternally expressed imprinted genes, *Peg3* and *Peg10*, predicted to increase the size of this compartment (John, 2013). This convergence suggests that this lineage is a major site of parental genomic conflict. The spongiotrophoblast is a major site of production of placental lactogens (Simmons et al., 2008). Some members of this extensive gene family (*Prl3d* and *Prl3b1*) have been shown to induce the changes in the mother required for a successful pregnancy (Bhattacharyya et al., 2002, Muller et al., 1999). The spongiotrophoblast also manufactures pregnancy-specific glycoproteins (PSGs) which are another family of highly similar secreted proteins thought to contribute in the protection of the semiallotypic fetus from the maternal immune system, and which also remodel placental and maternal vasculature (Kammerer and Zimmermann, 2010, Wu et al., 2008). Essentially the spongiotrophoblast is a hormone factory and the considerable drain on maternal resources required to sustain the production of these placental hormones may be why this lineage is so tightly regulated by imprinting. Alternatively, this regulation may reflect the function of these hormones in pregnancy. Placental hormones are manufactured in large quantities during pregnancy and act on the maternal system to direct resources to support fetal growth. While it remains to be determined whether such changes in gene expression in the placenta have a consequence for maternal physiology in this model, this data provides further evidence that the maternal and paternal genomes are involved in a continuing battle over the endocrine function of the mouse placenta.

We have previously shown that the maternally expressed *Phlda2* gene limits the expansion of the spongiotrophoblast alongside placental stunting, a loss of placental glycogen and fetal growth restriction (Tunster et al., 2010, Tunster et al., 2014, Tunster et al., 2015). Similarly, elevated *Ascl2* also resulted in fetal growth restriction but, in contrast to the *Phlda2* transgenic model, placental glycogen stores were markedly increased in the near term placenta when fetal growth restriction was apparent. Moreover, there was a marked appearance of clusters of glycogen cells in the labyrinth at a time when these cells are normally migrating into the decidua. Expression of some glycogen cell markers, such as the marker of migrating glycogen cells *Prl7b1*, was altered but the majority of markers were expressed at similar levels to WT suggesting mislocalisation from the junctional zone. A milder mislocalisation of junctional zone cells was also apparent in our *Phlda2* overexpression model where there was also a loss of spongiotrophoblast (Tunster et al., 2010) suggesting that the spongiotrophoblast is important for maintaining the glycogen cells within the junctional zone. Mislocalisation of glycogen cells in the Ascl2-Tg model was considerably more severe than in our *Phlda2* model suggesting that the loss of P-TGC may further contribute to this phenotype. The presence of excess glycogen and fetal growth restriction is not consistent with the suggestion that placental glycogen is required to support late fetal growth (Coan et al., 2006). However, these data can be reconciled if the release of glycogen into the maternal system was prevented as a consequence of the mislocalisation of glycogen cells into the labyrinth.

Fetal growth restriction was very modest (6%) on the BL6 background despite the rather dramatic loss of both P-TGC and spongiotrophoblast cells. BL6 placenta have a higher F:P ratio and are thought to have a greater reserve capacity to support fetal growth compared to the 129 strain. We had great difficulty breeding Ascl2-Tg into 129. Under standard conditions in a conventional unit few plugged females were pregnant at E14.5. When the line was rederived into a barrier unit where the mice were maintained on an enriched diet, the pregnancy success rate improved sufficiently to assess fetal growth but under these conditions we observed no fetal growth restriction. The use of different diets under a different health status confounds the interpretation of this data. Notably, targeted loss of function of the placental lactogens *Prl4a1* and *Prl7b1* had no overt phenotypic consequence under normal husbandry conditions but pregnancies failed under stressed conditions (Ain et al., 2004, Bu et al., 2016). There are 22 members of the *Prl* gene family (Simmons et al., 2008). These data suggests an inherent redundancy in the functions of members of the *Prl* family such that overt fetal complications manifest only when adverse conditions are combined with reduced expression.

Both loss-of-function (Guillemot et al., 1995) and overexpression of *Ascl2 in vivo* (Figs. 2D, and 5A) results in fewer cells expressing *Tpbpa*. Ectopic overexpression of *Ascl2* in TS cells also resulted in low *Tpbpa* (Takao et al., 2012). These data suggest that *Ascl2* is both required for the development of *Tpbpa*+ve lineages and restrains their proliferation. In the *Del*^*7A1/+*^ model, in which placenta express *Ascl2* at 50% normal, at E9.5 placenta initially appeared to have an increased number of cells expressing *Tpbpa*. By E15.5 very few cells expressed this marker (Oh-McGinnis et al., 2011). The *Del*^*7A1/+*^ model involves a 280 kb deletion of the IC1–IC2 interval directly or indirectly disrupting the expression of three imprinted genes in this region: *Ascl2* (50%), *Phlda2* (200%) and *Th* (0%) (Oh-McGinnis et al., 2011). We have previously shown that just two-fold expression of *Phlda2* alone can reduce the size of the spongiotrophoblast lineage by 50% (Tunster et al., 2010, Tunster et al., 2014, Tunster et al., 2015). The findings in these different models can be reconciled if the spongiotrophoblast phenotype in the *Del*^*7A1/+*^ model is due to elevated *Phlda2* rather than a direct consequence of elevated *Ascl2*.

Previous studies have suggested a direct relationship between *Ascl2* and *Phlda2* (Data summarised in Supplemental Table 1). *Ascl2* is co-expressed with *Phlda2* in a subset of cells in the ectoplacental cone at E7.5, E9.5 (Oh-McGinnis et al., 2011, Takao et al., 2012) and E10.5 (Fig. 7A) where progenitors of the spongiotrophoblast reside. Adenoviral-driven overexpression of *Ascl2* in trophoblast stem cells resulted in a 60% reduction in the expression of *Phlda2* under stem cell culture conditions (Takao et al., 2012). Conversely, knockdown of *Ascl2* expression in TS cells (Takao et al., 2012) or reduced expression *in vivo* (Oh-McGinnis et al., 2011) resulted in increased *Phlda2* expression. In this current study, we did not observe a statistically significant reduction in *Phlda2* expression *in vivo* in response to overexpression of *Ascl2*. When we genetically tested the relationship between *Ascl2* and *Phlda2* by combining overexpression of *Ascl2* with loss-of-expression of *Phlda2,* this resulted in a markedly expanded spongiotrophoblast similar to loss of expression of *Phlda2* alone. This tells us that, while both elevated *Phlda2* (Tunster et al., 2010, Tunster et al., 2014, Tunster et al., 2015) and elevated *Ascl2* repress the spongiotrophoblast, *Ascl2* can only do so in the presence of *Phlda2* (Fig. 7C). These data are consistent with *Ascl2* functioning upstream of *Phlda2* to control the expansion of the spongiotrophoblast in a progenitor cell type (Fig. 8).

In conclusion, we have demonstrated that overexpression of the imprinted *Ascl2* gene has considerable consequences for placental development, specifically for the P-TGC and spongiotrophoblast lineages both of which express pregnancy-related hormones. Either as a consequence of the reduced function of the endocrine compartment or the failure in the appropriate migration of glycogen cells, elevated *Ascl2* resulted in a late fetal growth restriction. The presence of three imprinted genes within a single mechanistically distinct imprinted domain that all act to regulate placental lineages critical for the endocrine function of the placenta suggest that the imprinting of this domain was key to the switch to prolonged gestation and greater maturity at birth observed in Eutherian mammals.

## Competing interests statement

The authors declare that there is no conflict of interest financial or otherwise associated with this submission.

## Author contributions

RMJ and SJT conceived and designed the experiments, interpreted the data and wrote the paper. SJT performed most of the experimental work; GIM generated material and performed dissections and HDJC supporting image capture and analysis.
