# Supplementary material for: Increased dosage of the imprinted Ascl2 gene restrains two key endocrine lineages of the mouse Placenta
Source: Dev Biol. 2016 Oct 1;418(1):55–65. doi: 10.1016/j.ydbio.2016.08.014 (PMC5040514; doi:10.1016/j.ydbio.2016.08.014)
Supplement: Supplementary file 1 — Supplementary material [file mmc1.pdf]

Supplemental Table 1: Summary of expression data expressed as % wild type levels from models in which expression of *Ascl2* has been manipulated (grey column).

System	Intervention	Sample	*Ascl2*	*Phlda2*	*Prl3d*	*Tpbpa*	Ref
Rcho-1	CMV-driven/transient tranfection	Rcho-1	n.d.	n.d.	Down	n.d.	[24]
Rcho-1	CMV-driven/transient tranfection	Rcho-1	n.d.	n.d.	**Down*	n.d.	[25]
Rcho-1	CMV-driven/transient tranfection	Rcho-1	n.d.	n.d.	***No change*	n.d.	[26]
TS cells	Adenoviral CMV overexpression of *Ascl2*	TS cells	10X	40%	70%	70% (n.s.)	[27]
Mouse model	Ascl2-Tg	E9.5 placenta	6X	80% (ns)	75%	70% (n.s.)	Fig 2 and 7
Mouse model	Ascl2-Tg	E14.5 placenta	6X	80% (n.s.)	n.d.	40%	Fig 5 and 7
TS cells	siRNA *Ascl2*	TS cells	15%	4X	200% (n.s.)	240%	[27]
Mouse model	*Ascl2*$^{-/+}$	E8.5 placenta	0%.	n.d.	Up	Very little expression (in situ)	[8]
Mouse model	Ascl2-lacZ allele	E9.5 placenta	5%	115%	Up	DOWN	[17]
Mouse model	*Del7AI* (280 kb deletion spanning *Th)*	E9.5 placenta	50%	200%	Up	150% (n.s.)	[17]
Mouse model	*Del7AI* (280 kb deletion spanning *Th)*	E15.5 placenta	40%	150%	n.d.	Very little expression (in situ)	[17]

n.d. = no data; n.s. = not significant; * Presumed down based on cell morphology; ** presumed no change based on cell morphology

Supplemental Table 2: QPCR primer sequences

Gene	Primer 1	Primer 2
Blimp1	GGGTACTTCTGTTCAAGCCG	TCCTGTTGGCATTCTTGGGA
Rgs5	TGAGAAGCCAGAGAAGCCTG	GCCTTCTCCGCCATTTTGAT
Pcsk6	GATATGACGCCAGCAACGAG	CCAACTGGCGCTGTAAATGT
Tpbpb	AAGCAACGTGGAATGAGTTTG	TCCAACATTGGGTGGAAAAT
Prl3c1	TGGGATGATGACAAGAGCTG	GCAAGAAGGTGCTTGCTTTT
Prl7a2	ATCCATCAATGGGCTGTTTC	AGGTGAGAGCCTTGACCAGA
Prl8a9	ATTCCTGCATGTATGGCACA	CAGCTCTGGCAACAGTCTCA
Psg17	CCCTTCGAACCGTAAGTCAA	CACAACTGCTCCTTTGTACCAG
Psg21	TTGCTGGAGCTGAAGGTTTT	CGTGTTTTGGTGACTGGATG
Prl8a1	ACAGATGAGGAAGGCTGCAT	TGGCTCTGAGAACAAGCTGA
Prl8a6	GTTGTGGGAAAAAGCTGCAT	TCCCAAAATTGTCCAGATGAG
Prl7b1	CAGCACATCAATAGCCTTGC	TTGGTGATTTGAGTGGCAAA
Prl6a1	TGCTGTTGGCATCAAACTTC	TGCCTGGTTTATATCGCACA
Prl2a1	TCCAAAACCAGGGAGTCAAG	ACTCCCTCCAGGAGTCCATT
Tfeb	GCGGCAGAAGAAAGACAATC	CTGCATCCTCCGGATGTAAT
Synb	CTGGCACTTCATTCCCATTT	TGGCTGTAGGCTCTCAGGTT

Supplemental Table 3: Data for Figure 1

	E9.5	E10.5	E12.5
Fold change and *p* value	6.52 ± 2.31<br>$p = 0.0214$	6.14 ± 0.97<br>$p = 3.04 \times 10^{-4}$	6.17 ± 1.58<br>$p = 2.84 \times 10^{-3}$

Supplemental Table 4: Data for Figure 2

C: Giant Cell Count		
36.3 ± 7.1 versus 19.8 ± 1.9, n = 26; $p = 0.0120$		
D: Giant Cell Markers		
	E9.5	E10.5
Prl3d	0.73 ± 0.08<br>$p = 0.0468$	0.69 ± 0.09<br>$p = 0.0418$
Prl3b1	0.31 ± 0.18<br>$p = 0.0671$	0.60 ± 0.09<br>$p = 0.0243$
Prl2c	0.85 ± 0.12<br>$p = 0.305$	0.67 ± 0.12<br>$p = 0.0961$
Hand1	0.59 ± 0.34<br>$p = 0.414$	0.63 ± 0.12<br>$p = 0.0934$
E: Early differentiation markers		
	E9.5	E10.5
Tpbpa	0.73 ± 0.22<br>$p = 0.380$	0.75 ± 0.20<br>$p = 0.378$
Blimp1	1.20 ± 0.15<br>$p = 0.182$	1.20 ± 0.18<br>$p = 0.241$
Rgs5	0.97 ± 0.12<br>$p = 0.846$	1.03 ± 0.18<br>$p = 0.887$
Pcsk6	1.03 ± 0.18<br>$p = 0.854$	0.94 ± 0.16<br>$p = 0.739$
Prl7b1	1.12 ± 0.36<br>$p = 0.741$	1.78 ± 0.67<br>$p = 0.127$
Pcdh12	1.23 ± 0.26<br>$p = 0.369$	1.52 ± 0.20<br>$p = 0.0176$

Supplemental Table 5: Data for Figure 3

	Area (mm2)					
	**Jz**	**Lab**	**Total**	**Number of clusters**	**Total area (mm2)**	**Average area (mm2)**
**WT**	3.58 ± 0.22<br>n = 3	4.81 ± 0.59<br>n = 3	8.40 ± 0.81<br>n = 3	3 ± 1.53<br>n = 3	0.058 ± 0.07<br>n = 3	0.013 ± 0.0025<br>n = 3
**Tg**	2.24 ± 0.29<br>n = 4	5.24 ± 0.53<br>n = 4	7.48 ± 0.51<br>n = 4	20 ± 3.09<br>n = 4	0.272 ± 0.06<br>n = 4	0.013 ± 0.0021<br>n = 4
**Ratio and *p* value**	62.6%<br>p = 0.0185	108.9%<br>p = 0.616	89.1%<br>p = 0.359	675%<br>p = 0.00670	467%<br>p = 0.0442	102.5%<br>p = 0.146

Supplemental Table 6: Data for Figure 4

	Area (mm2)		
	**Jz**	**Lab**	**Total**
**WT**	1.74 ± 0.20<br>n = 8	5.10 ± 0.17<br>n = 8	6.83 ± 0.33<br>n = 8
**Tg**	1.52 ± 0.12<br>n = 7	5.65 ± 0.09<br>n = 7	7.18 ± 0.19<br>n = 7
**Ratio and *p* value**	87.6%<br>p = 0.393	110.9%<br>p = 0.0153	105.0%<br>p = 0.410

Supplemental Table 7: Data for Figure 5

A: Junctional zone	
Tpbpa	0.43 ± 0.07<br>$p = 5.14 \times 10^{-3}$
Tpbpb	0.25 ± 0.06<br>$p = 1.93 \times 10^{-3}$
Flt1	0.37 ± 0.07<br>$p = 4.74 \times 10^{-3}$
B: Spongiotrophoblast	
Prl8a8	0.16 ± 0.04<br>$p = 3.95 \times 10^{-4}$
Prl3a1	0.11 ± 0.04<br>$p = 8.04 \times 10^{-3}$
Prl3c1	0.21 ± 0.06<br>$p = 1.67 \times 10^{-3}$
Prl7a2	0.41 ± 0.11<br>$p = 0.0335$
Prl3b1	0.44 ± 0.08<br>$p = 1.57 \times 10^{-3}$
Prl8a9	0.32 ± 0.06<br>$p = 1.69 \times 10^{-3}$
Psg17	0.16 ± 0.04<br>$p = 6.10 \times 10^{-4}$
Psg18	0.48 ± 0.12<br>$p = 8.43 \times 10^{-3}$
Psg19	0.23 ± 0.05<br>$p = 2.25 \times 10^{-4}$
Psg21	0.26 ± 0.08<br>$p = 6.42 \times 10^{-3}$
Prl8a1	0.88 ± 0.18<br>$p = 0.555$
Prl8a6	0.92 ± 0.09<br>$p = 0.417$

C: Glycogen Cells	
Pcdh12	1.25 ± 0.15<br>$p = 0.103$
Gjb3	0.85 ± 0.07<br>$p = 0.0818$
Prl7b1	3.17 ± 0.72<br>$p = 8.73 \times 10^{-3}$
Prl6a1	2.99 ± 1.09<br>$p = 0.107$
Prl2a1	2.23 ± 0.38<br>$p = 0.00569$
Gbe1	1.54 ± 0.12<br>$p = 0.00308$
Gyg	0.87 ± 0.13<br>$p = 0.352$
Gys	0.86 ± 0.11<br>$p = 0.260$
Ugp2	0.87 ± 0.06<br>$p = 0.0902$
D: Giant Cells	
Hand1	0.73 ± 0.15<br>$p = 0.156$
Ctsq	0.72 ± 0.11<br>$p = 0.102$
Prl2c	0.95 ± 0.12<br>$p = 0.733$
Tle3	0.78 ± 0.13<br>$p = 0.163$
E: Labyrinth	
Flk1	0.79 ± 0.14<br>$p = 224$
Gcm1	1.23 ± 0.43<br>$p = 0.605$
Syna	0.88 ± 0.25<br>$p = 0.664$
Dlx3	0.95 ± 0.21<br>$p = 0.819$
Tfeb	0.88 ± 0.18<br>$p = 0.580$
Ly6e	1.02 ± 0.16<br>$p = 0.921$
Synb	1.11 ± 0.26<br>$p = 0.672$
Cebpa	0.99 ± 0.16<br>$p = 0.955$

Supplemental Table 8: Data for Figure 6

	A: Fetal weight (g)			
	E12.5	E14.5	E16.5	E18.5
WT	0.086 g ± 0.002 n = 29	0.244 g ± 0.003 n = 42	0.659 g ± 0.009 n = 25	1.209 g ± 0.010 n = 41
Tg	0.090 g ± 0.003 n = 19	0.241 g ± 0.005 n = 40	0.644 g ± 0.011 n = 28	1.135 g ± 0.026 n = 33
Ratio and *p* value	104.4% *p* = 0.264	98.9% *p* = 0.660	97.7% *p* = 0.280	93.9% *p* = 0.00521
	B: Placental weight (mg)			
WT	64.8 mg ± 2.41 n = 29	99.8 mg ±1.62 n = 42	106.3 mg ± 1.73 n = 25	98.7 mg ± 1.10 n = 41
Tg	54.0 mg ± 3.21 n = 19	88.8 mg ± 2.12 n = 40	101.8 mg ± 2.58 n = 28	97.3 mg ± 1.95 n = 33
Ratio and *p* value	83.4% *p* = 0.00898	89.1% $p = 9.29 \times 10^{-5}$	95.7% *p* = 0.162	98.6% *p* = 0.511
	C: F:P Ratio			
WT	1.4 ± 0.06 n = 29	2.5 ± 0.03 n = 42	6.2 ± 0.11 n = 25	12.3 ± 0.14 n = 41
Tg	1.8 ± 0.11 n = 19	2.7 ± 0.07 n = 40	6.4 ± 0.18 n = 28	11.7 ± 0.22 n = 33
Ratio and *p* value	127.5% *p* = 0.00130	111.9% $p = 1.81 \times 10^{-4}$	103.1% *p* = 0.373	95.2% *p* = 0.0201

	D: Glycogen (mg)		
	E14.5	E16.5	E18.5
WT	1.23 mg ± 0.11 n = 8	1.17 mg ± 0.08 n = 13	0.44 mg ± 0.03 n = 14
Tg	1.08 mg ± 0.07 n = 10	1.44 mg ± 0.09 n = 23	0.71 mg ± 0.08 n = 8
Ratio and *p* value	87.7% *p* = 0.260	122.8% *p* = 0.0598	161.4% *p* = 0.00136
	E: Glycogen (mg/g)		
WT	12.10 mg ± 0.99 n = 8	11.27 mg ± 0.85 n = 13	4.63 mg ± 0.33 n = 14
Tg	12.35 mg ± 0.69 n = 10	14.12 mg ± 0.91 n = 23	7.14 mg ± 0.79 n = 8
Ratio and *p* value	102.0% *p* = 0.836	125.2% *p* = 0.0458	154.2% *p* = 0.00277

Supplemental Table 9: Mating success rate on 129 background examined at E14.5

Strain	Diet	Number of plugs	% Pregnant at E14.5	Average number of fetuses (when pregnant)	Total number of fetuses
129	Lab Diet 5008	10	90%	6.2	56
129	SDS RM3	10	50%	4.	20

Supplemental Table 10: Fetal weight data for 129 strain at E18.5

	**129**
**WT**	1.14 ± 0.006 n = 51
**Tg**	1.12 ± 0.012 n = 50
**Tg vs WT Ratio and *p* value**	98.1% *p* = 0.299
