# Supplementary figures and images for: Increased dosage of the imprinted Ascl2 gene restrains two key endocrine lineages of the mouse Placenta
Source: Dev Biol. 2016 Oct 1;418(1):55–65. doi: 10.1016/j.ydbio.2016.08.014 (PMC5040514; doi:10.1016/j.ydbio.2016.08.014)

Supplemental Table 1: Expression of *Ascl2* relative to *Tspan32* in wild type and *Ascl2*-Tg placenta

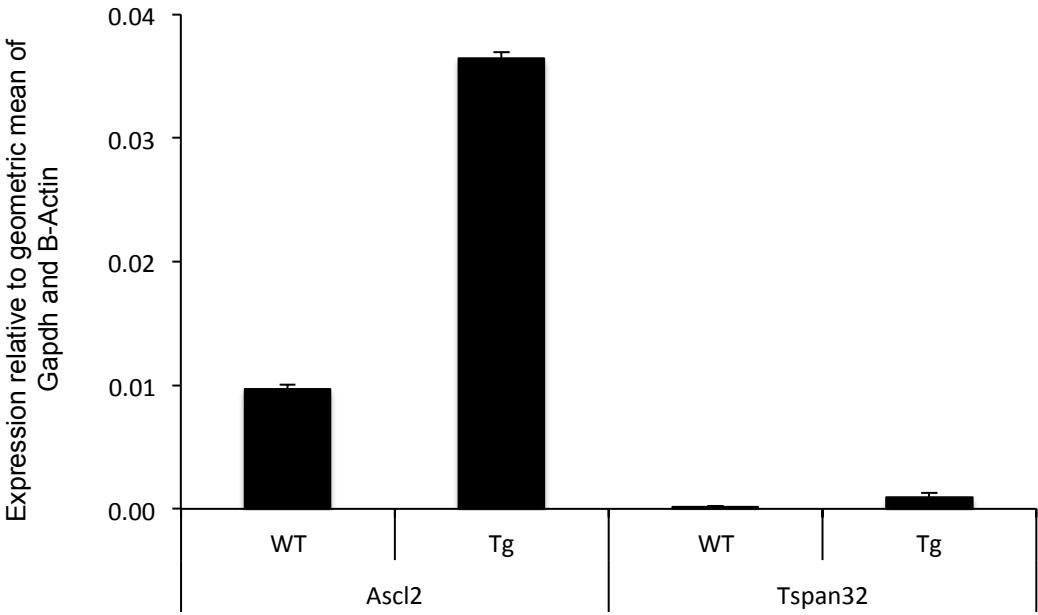

Supplement: Supplementary file 2 — Supplementary material [file mmc2.pdf]
